# Supplementary material for: Compressive three-dimensional super-resolution microscopy with speckle-saturated fluorescence excitation
Source: Nat Commun. 2019 Mar 22;10:1327. doi: 10.1038/s41467-019-09297-5 (PMC6430798; doi:10.1038/s41467-019-09297-5)
Supplement: Supplementary file 1 — Supplementary Information [file 41467_2019_9297_MOESM1_ESM.pdf]

Supplementary Information

Pascucci et al.

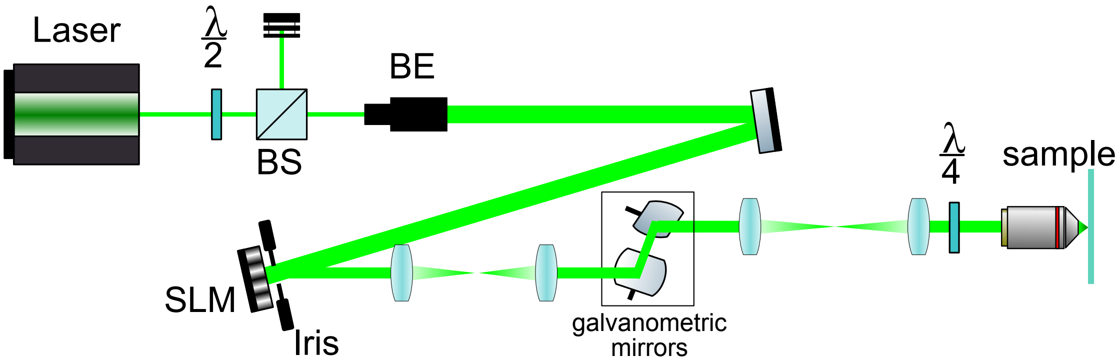

Supplementary Figure 1 | Complete scheme of the speckle scanning microscope. The laser power is modulated using a half-wave plate ($\lambda/2$) and a polarizing beam splitter (BS). The laser beam then passes through a beam-expander (BE) before illuminating the spatial light modulator (SLM) which generates the speckle. The SLM is conjugated to a pair of galvanometric mirrors and to the back focal plane of the microscope objective. A quarter wave-plate ($\lambda/4$) circularly polarizes the impinging beam in order to achieve isotropic transverse super-resolution.

Supplementary Note 1: Optical Transfer Function (OTF) of a scanning Microscope

Here we derive the two-dimensional optical transfer function (OTF) of a speckle scanning microscope in the linear (i.e. non-saturated) excitation regime and demonstrate that the speckle OTF better probes the high spatial frequencies, especially in the vicinity of the OTF support boundary.

Imaging system

The two-dimensional image I obtained by an optical system of a object consisting in a spatial density of fluorophore O is given by a convolution with the system point spread function PSF:

$$I(\mathbf{r}) = \int O(\mathbf{r}')\mathrm{PSF}(\mathbf{r}-\mathbf{r}')d\mathbf{r}' \quad (1)$$

$$= O * \mathrm{PSF} \quad (2)$$

Fourier transforming the former equation gives:

$$\mathscr{F}(I) = \mathscr{F}(O)\mathscr{F}(\mathrm{PSF}) \quad (3)$$

where $\mathscr{F}(\mathrm{PSF})$ is called the optical transfer function (OTF). The OTF thus filters the object by modulating the amplitude of its Fourier components and even canceling those lying outside the OTF support. Ideally, for the optical system to be as reliable as possible, the OTF should then be as flat as possible, at least over its support.

In the following, we compare the two-dimensional OTF of a point-scanning microscope and of a speckle-scanning microscope.

OTF of a point-scanning microscope

The PSF of a non-aberrant point-scanning microscope PSF_0 is given by the (inverse) Fourier transform of the objective lens pupil Π:

$$\mathrm{PSF}_0 = |\mathscr{F}^{-1}(\Pi)|^2 \quad (4)$$

and the corresponding OTF is then:

$$\mathrm{OTF}_0 = \mathscr{F}\left[\mathscr{F}^{-1}(\Pi)\mathscr{F}^{-1,*}(\Pi^*)\right] \quad (5)$$

$$= \Pi \star \Pi^* \quad (6)$$

where $\star$ designate the cross-correlation product.

We now assume that the objective lens pupil is real and that it has simply a disk-shaped profile ($\Pi = 1$ over this disk and 0 outside). In the spatial-frequency domain, the radius of the disk is given by the numerical aperture (NA) of the objective lens and is equal to NA/λ. The OTF being the cross-correlation product of this disk, the OTF profile is thus just given by the overlapping area of two disks of same radius which can be trivially analytically derived as:

$$\mathrm{OTF}_0(\mathbf{w}_\perp) = 2\left(\frac{\mathrm{NA}}{\lambda}\right)^2\left[\arccos\left(\frac{w_\perp\lambda}{2\mathrm{NA}}\right) - \frac{w_\perp\lambda}{2\mathrm{NA}}\sqrt{1-\left(\frac{w_\perp\lambda}{2\mathrm{NA}}\right)^2}\right] \quad (7)$$

The normalized plot of this profile is shown in Supplementary Fig. 2. The OTF vanishes at twice the pupil disk radius, namely $2\mathrm{NA}/\lambda$.

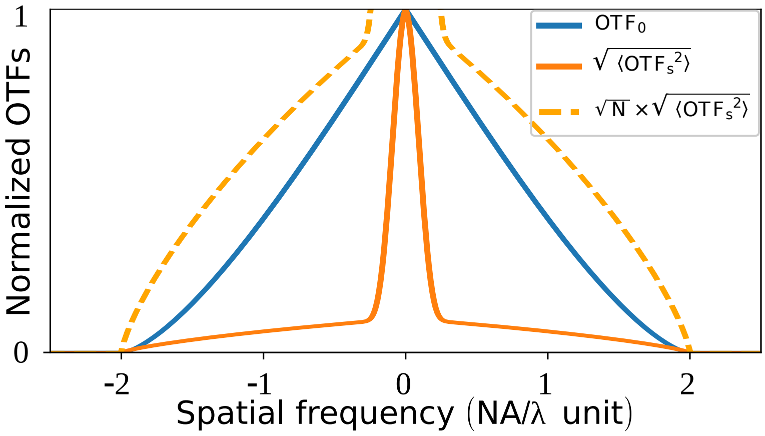

Supplementary Figure 2 | Comparison of Optical Transfer Functions. Optical transfer function of a point scanning microscope (OTF_0) and the mean squared OTF of a speckle scanning microscope (OTF_s). Above the low-frequency peak (corresponding to the inverse of the speckle spot), the speckle scanning OTF is proportional to the square root of OTF_0, ensuring more efficient probing of high spatial frequencies.

OTF of a speckle-scanning microscope

In our experiment, the speckle is generated by a spatial light modulator conjugated to the back focal plane of the objective lens. More generally, a speckle can be typically obtained by placing a random phase mask at the back focal plane of a lens. The field at the pupil plane may then be written $\Pi e^{i\varphi(\mathbf{w}_\perp)}$ where Π is the former pupil profile and φ a random phase characterized by the covariance:

$$\left\langle e^{i\varphi(\mathbf{w}_\perp)} e^{-i\varphi(\mathbf{w}'_\perp)} \right\rangle = g\left(\frac{\|\mathbf{w}_\perp - \mathbf{w}'_\perp\|}{w_0}\right) \tag{8}$$

where $\langle\cdot\rangle$ designates the statistical average, g is the covariance function of the field and w_0 the correlation width of the random field in the pupil plane. The covariance function is peaked at value $\mathbf{w} = \mathbf{0}$ ($g(0) = 1$), vanishes for large frequencies, and is characterized by a profile-width w_0. w_0 actually dictates the size of the speckle spot at the sample plane as illustrated in Supplementary Figs. 3. The spectral extent of the pupil support being NA/λ, we define w_0 so that the number of speckle grains is:

$$N = \left(\frac{\mathrm{NA}}{\lambda w_0}\right)^2 \tag{9}$$

Assuming N is large, the statistically averaged OTF of a speckle-scanning microscope, obtained by calculating the cross-correlation product of $\Pi e^{i\varphi}$ with itself, is then trivially:

$$\langle \mathrm{OTF}_s \rangle (\mathbf{w}_\perp) = g\left(\frac{w_\perp}{w_0}\right) \mathrm{OTF}_0(\mathbf{w}_\perp) \tag{10}$$

This average thus vanishes for frequencies larger than w_0 which means that the imaging resolution is limited by the size of the speckle spot ($1/w_0$) at the sample plane. However, prior characterization of the speckle point spread function provides the knowledge of the complex-valued OTF. Statistical complex-averaging of the OTF thus does not make sense in this case.

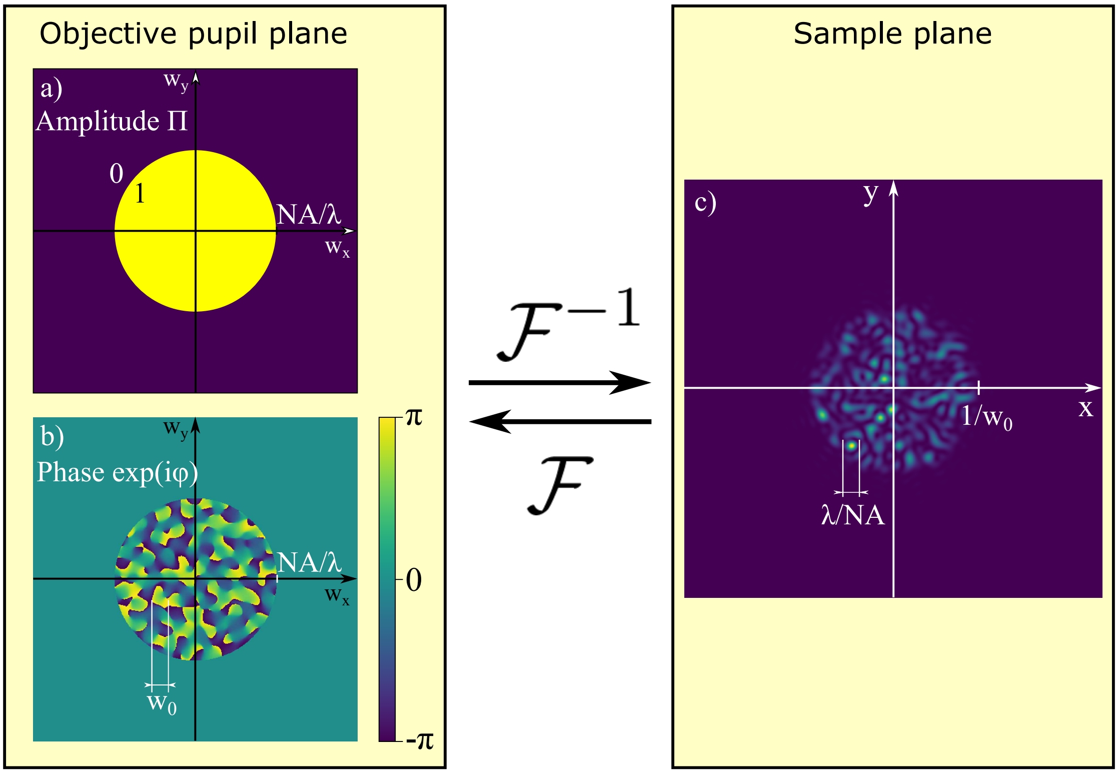

Supplementary Figure 3 | Notations for the calculation of OTFs. In the back focal plane of the objective lens, the amplitude is assumed to be binary disk-shaped (a). The spatial frequency at the pupil boundary is NA/λ. For speckle projection, a random phase is added (b). The correlation width of the field is notated w_0. The field at the sample plane is given by an inverse Fourier transform relation relatively to the pupil plane of the objective. The width of the resulting speckle spot (c) is $1/w_0$ and the speckle grain size is λ/NA.

The relevant average to calculate is then $\sqrt{\langle |OTF_s|^2 \rangle}$. The mean squared value of the OTF gives information about how the sample frequencies are probed by the speckle pattern. $\langle |OTF_s|^2 \rangle$ can be calculated the following way:

$$\begin{aligned}&\langle |OTF_s|^2 \rangle \\ &= \iint \Pi(\mathbf{w_1})\Pi(\mathbf{w_2})\Pi(\mathbf{w_1}+\mathbf{w})\Pi(\mathbf{w_2}+\mathbf{w}) \left\langle e^{-i\varphi(\mathbf{w_1})} e^{i\varphi(\mathbf{w_2})} e^{i\varphi(\mathbf{w_1}+\mathbf{w})} e^{-i\varphi(\mathbf{w_2}+\mathbf{w})} \right\rangle d\mathbf{w_1} d\mathbf{w_2}\end{aligned} \tag{11}$$

According to the moment theorem for zero-mean Gaussian processes [1], the statistical average in the integral can be simplified provided that:

$$\langle Z_1 Z_2^* Z_3 Z_4^* \rangle = \langle Z_1 Z_2^* \rangle \langle Z_3 Z_4^* \rangle + \langle Z_1 Z_4^* \rangle \langle Z_3 Z_2^* \rangle \tag{12}$$

thus yielding:

$$\begin{aligned}\left\langle e^{-i\varphi(\mathbf{w_1})}e^{i\varphi(\mathbf{w_2})}e^{i\varphi(\mathbf{w_1}+\mathbf{w}_\perp)}e^{-i\varphi(\mathbf{w_2}+\mathbf{w}_\perp)}\right\rangle &= \left\langle e^{-i\varphi(\mathbf{w_1})}e^{i\varphi(\mathbf{w_2})}\right\rangle \left\langle e^{i\varphi(\mathbf{w_1}+\mathbf{w}_\perp)}e^{-i\varphi(\mathbf{w_2}+\mathbf{w}_\perp)}\right\rangle \\ &+ \left\langle e^{-i\varphi(\mathbf{w_1})}e^{i\varphi(\mathbf{w_1}+\mathbf{w}_\perp)}\right\rangle \left\langle e^{i\varphi(\mathbf{w_2})}e^{-i\varphi(\mathbf{w_2}+\mathbf{w}_\perp)}\right\rangle \\ &= g^2\left(\frac{\|\mathbf{w}_1-\mathbf{w}_2\|}{w_0}\right) + g^2\left(\frac{\|\mathbf{w}_\perp\|}{w_0}\right) \qquad (13)\end{aligned}$$

and assuming that $N \gg 1$, Eq. (11) can be simplified into:

$$\left\langle |OTF_s|^2 \right\rangle(\mathbf{w}_\perp) = g^2\left(\frac{\|\mathbf{w}_\perp\|}{w_0}\right)\mathrm{OTF}_0^2(\mathbf{w}_\perp) + \left[\int g^2\left(\frac{\|\mathbf{w}\|}{w_0}\right)d\mathbf{w}\right]\mathrm{OTF}_0(\mathbf{w}_\perp) \qquad (14)$$

At zero frequency, where OTF_0 is maximum, and assuming that $g(\mathbf{u}) = e^{-\mathbf{u}^2/2}$, the first term is equal to $\left[\pi\left(\frac{NA}{\lambda}\right)^2\right]^2$ while the second one is equal to $\left(\pi w_0^2\right)\left[\pi\left(\frac{NA}{\lambda}\right)^2\right] = \frac{1}{N}\left[\pi\left(\frac{NA}{\lambda}\right)^2\right]^2$, a factor N smaller. At low spatial frequencies (smaller than w_0), $\sqrt{\langle |OTF_s|^2\rangle}$ can then be approximated by:

$$\begin{aligned}\sqrt{\langle |OTF_s|^2\rangle} &\simeq g\left(\frac{w_\perp}{w_0}\right)\mathrm{OTF}_0(\mathbf{w}_\perp) \qquad (15)\\ &\simeq \langle \mathrm{OTF}_s\rangle \qquad (16)\end{aligned}$$

This profile correspond to the central peak of the solid orange line in Supplementary Fig. 2. For frequencies larger than w_0, where g vanishes:

$$\begin{aligned}\sqrt{\langle |OTF_s|^2\rangle} &\simeq \left\{\left[\int g^2\left(\frac{\|\mathbf{w}\|}{w_0}\right)d\mathbf{w}\right]\mathrm{OTF}_0(\mathbf{w}_\perp)\right\}^{1/2} \qquad (17)\\ &\simeq \sqrt{\pi}w_0\sqrt{\mathrm{OTF}_0(\mathbf{w}_\perp)} \qquad (18)\end{aligned}$$

This profile is illustrated by the large side-lobes of orange curves in Supplementary Fig. 2. Since the peak amplitude of this profile is a factor N as small as the central peak as explained above, the N-times magnified profile is shown in Supplementary Fig. 2 as the dotted orange line. Since OTF_0 vanishes linearly at its support boundary $w \leq \frac{2\mathrm{NA}}{\lambda}$, the speckle OTF exhibits vertical asymptotes at this boundary, ensuring more efficient probing of the object frequencies.

Supplementary Note 2: Orthogonality of independent speckle patterns

Two speckle intensity appearing at different axial planes are statistically independent if the separation distance between the two planes is larger than $\simeq 2n\lambda/\mathrm{NA}^2$. The cross-correlation of two independent zero-mean random processes is zero. Speckled intensity are not zero-mean but the mean value can be easily removed. Based on the cross-correlation product, we may then use the following inner product for speckles [2]:

$$\langle S_1, S_2\rangle = (S_1 - \langle S_1\rangle) \star (S_2 - \langle S_2\rangle)(0) \qquad (19)$$

This inner product is zero for two independent speckles and for $S_1 = S_2$, $\langle S_1, S_2\rangle = \left\langle (S_1 - \langle S_1\rangle)^2\right\rangle$. Two independent speckle are then uncorrelated according to this inner product defined from the cross-correlation product and may be said "orthogonal". The inner product can be calculated in the Fourier domain:

$$\langle S_1, S_2\rangle = \mathscr{F}^{-1}\left\{\left[\mathscr{F}(S_1) - \mathscr{F}(S_1)(0)\right]^*\left[\mathscr{F}(S_2) - \mathscr{F}(S_2)(0)\right]\right\} \qquad (20)$$

Supplementary Note 3: Similarities beween Wiener deconvolution and cross-correlation

Wiener deconvolution is typically performed in the Fourier domain. Assuming the OTF of the imaging system is known, Wiener-deconvolution uses the following kernel:

$$K = \frac{\mathrm{OTF}^*}{|\mathrm{OTF}^2| + \sigma^2} \tag{21}$$

where σ prevents division by zero at locations where the OTF is smaller than the noise level or even vanishes. The restored object $\hat{O}$ is then obtained by:

$$\hat{O} = \mathscr{F}^{-1}\left[K\mathscr{F}(I)\right] \tag{22}$$

Deconvolution is then very similar to a cross-correlation product. The single difference is the amplitude renormalization at the denominator of K in Eq. (21). Two speckles that are orthogonal with respect to the cross-correlation product are thus orthogonal with respect to deconvolution also.

Supplementary Note 4: Sparsity requirements for 3D imaging by Wiener deconvolution of a 2D scan

The speckle pattern at the sample plane has a correlation length of the order of $\delta z = \frac{2n\lambda}{\mathrm{NA}^2}$ along the propagation axis, which means that two speckle further away than this distance are orthogonal with respect to the cross-correlation product. Moreover, the speckle grain size remains invariant over a axial range of the order of $\Delta z = 2Rn/\mathrm{NA}$, where $R = \sqrt{N}\frac{\lambda}{NA}$ is the radius of the speckle spot. Consequently, the number of independent axial planes in this range is $N_z = \frac{\Delta z}{\delta z} = \sqrt{N}$. In the manuscript body, we deconvolve a two-dimensional scan image by every individual speckle slice of the three-dimensional speckle point spread function. Only if a point source is in this slice deconvolution yields a bright point (illustrated in Supplementary Fig. 4c), otherwise only noise is obtained (illustrated in Supplementary Fig. 4). A two-dimensional scan then seems to paradoxically provide the ability to image a volume with $N^{3/2}$ speckle grains. In the following we demonstrate that a two-dimensional scan can indeed provide a three-dimensional representation of an object, under sparsity assumptions.

To estimate the required degree of sparsity, let's consider a point source of same brightness located at two different axial positions along the optical axis. A two-dimensional scanning of these fluorescent probes with a speckle PSF will result in the incoherent sum of two independent speckles S_1 and S_2 of same statistical properties (illustrated in Supplementary Figs. 4a and 4b). Deconvolution based on S_1 will result in a bright spot of size λ/NA and of amplitude close to one for the first fluorescent point source (Supplementary Fig. 4c). The peak amplitude depends on the noise parameter σ chosen for deconvolution. For the second point source, deconvolution will result into noise spreading over a surface $\frac{\pi}{w_0^2} = N\pi\left(\frac{\lambda}{\mathrm{NA}}\right)^2$ (Supplementary Fig. 4d). The image reconstruction will then result in a limited signal to noise ratio defined by the signal fluctuations resulting from the deconvolution of S_2 by S_1.

To estimate this signal to noise ratio, we approximate deconvolution by a cross-correlation product. Choosing for the sake of simplicity $\langle S_1\rangle = \langle S_2\rangle = 1$ over the speckle spot dimension, we write $S_i = 1 + s_i$ with $\langle s_i\rangle = 0$. For a fully developed speckle pattern with Gaussian statistics and Rayleigh intensity distribution, we also have $\left\langle s_i^2\right\rangle = 1$. Assuming that the number of speckle grains in the spot N is such that $N \gg 1$, reconstruction of point source 1 by cross-correlation

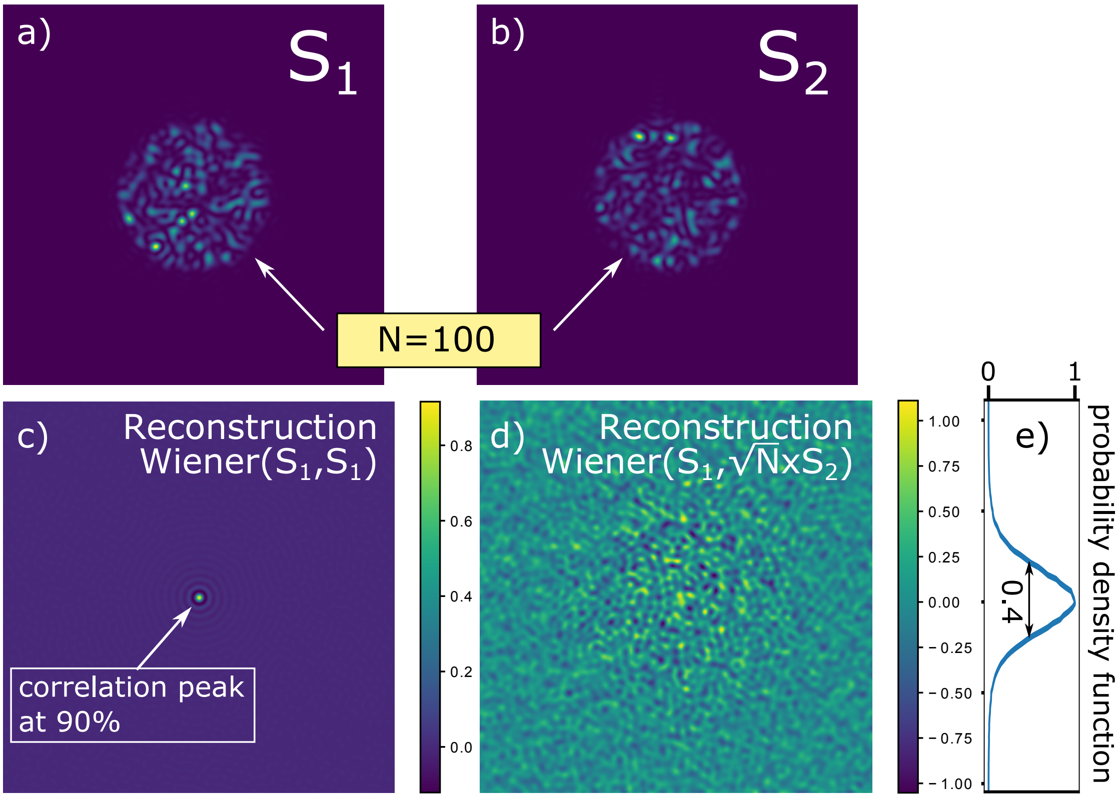

Supplementary Figure 4 | Signal to noise obtained by Wiener deconvolution. Illustration of deconvolution of two independent speckle spots (a and b) containing $N = 100$ speckle grains each. Wiener deconvolution of S_1 by itself yields a bright centered point (c). The noise parameter in the Wiener deconvolution process was set so that the point peak reaches 0.9. For the same noise parameter, deconvolution of S_2 by S_1 logically only results into noise whose amplitude depends on the relative average amplitude between S_1 and S_2 (d). For a S_2 average amplitude a factor $\sqrt{N}(= 10)$ as large as the one of S_1, the probability density function of the noise is 0.4 full width at half maximum (e).

with S_1 then yields a peak at origin with amplitude:

$$R_{11} = \frac{w_0^2}{\pi}\int_{\pi/w_0^2} S_1^2 d\mathbf{r} \tag{23}$$

$$= 2 \tag{24}$$

The cross-correlation of S_2 and S_1 yields a noisy background:

$$R_{21} = \frac{w_0^2}{\pi}\int_{\pi/w_0^2} S_1 S_2 d\mathbf{r} \tag{25}$$

$$= 1 + \frac{w_0^2}{\pi}\int_{\pi/w_0^2} (s_1 + s_2) d\mathbf{r} + \frac{w_0^2}{\pi}\int_{\pi/w_0^2} s_1 s_2 d\mathbf{r} \tag{26}$$

Statistical averaging just yields $R_{21} = 1$ which correspond to a constant background. Noise

must be estimated by calculating the second cumulant of R_{21}, resulting from the last integral in Eq. (26):

$$\left\langle \left(\frac{w_0^2}{\pi} \int_{\pi/w_0^2} s_1 s_2 d\mathbf{r} \right)^2 \right\rangle = \left(\frac{w_0^2}{\pi} \right)^2 \iint_{\pi/w_0^2} \langle s_1(\mathbf{r}) s_1(\mathbf{r}') \rangle \langle s_2(\mathbf{r}) s_2(\mathbf{r}') \rangle \, d\mathbf{r} d\mathbf{r}' \quad (27)$$

$$= \left(\frac{w_0^2}{\pi} \right)^2 \iint_{\pi/w_0^2} g^2 \left(\frac{\|\mathbf{r}-\mathbf{r}'\|}{\lambda/\mathrm{NA}} \right) d\mathbf{r} d\mathbf{r}' \quad (28)$$

$$= \left(\frac{\lambda w_0}{\mathrm{NA}} \right)^2 \quad (29)$$

$$= \frac{1}{N} \quad (30)$$

The cross-correlation of S_2 and S_1 thus results in noise of amplitude $1/\sqrt{N}$. This noise must be compared to the 1-amplitude peaked signal obtained by the cross-correlation of S_1 with itself (once the 1-background is removed).

In the linear excitation regime, 3D object reconstruction by plane-by-plane Wiener deconvolution of a two-dimensional scan requires that the number of point sources (of same brightness) is smaller than $\sqrt{N}$ in the speckle-PSF volume with N the number of speckle grains in the two-dimensional speckle PSF. Equivalently, if only two point sources of different brightness are in this volume, the dimer one can be reconstructed above the noise level if the brightness ratio between the two point sources is smaller than $\sqrt{N}$. To illustrate so, we plotted the Wiener deconvolution result of S_1 by S_1 (Supplementary Fig. 4c) and of S_1 by $\sqrt{N}S_2$ (Supplementary Fig. 4d) and plotted the histogram in Supplementary Fig. 4e. The noise histogram is centered at zero values (since speckles are orthogonal) but exhibit fluctuations of the of 1. Although the former analytical calculations were derived using the cross-correlation product and not Wiener deconvolution, the obtained results are qualitatively validated by numerical simulations.

Importantly, since the number of axial planes in the three-dimensional point spread function scales as $\sqrt{N}$, just like the required degree of sparsity, it must be pointed out that a line crossing the psf volume can be imaged with a signal to noise ratio equal to 1. Uniformly fluorescent single actin filaments crossing the PSF volume can the be reconstructed by Wiener deconvolution with a signal to noise ratio of 1.

Supplementary Note 5: 3D object reconstruction using Fast Iterative Shrinkage Thresholding Algorithm (FISTA)

Object reconstruction by Wiener deconvolution is not optimal at least for two reasons related to the statistical orthogonality of independent speckles: - out-of-focus objects yield noise in the plane of interest and - a single point-object reconstruction is associated with noise around. In this regard, a compressed sensing algorithm is optimized to avoid these drawbacks.

The Fast Iterative Shrinkage Thresholding Algorithm (FISTA) [3] is a well-known method to solve the basic linear inverse problem. It is a modification of the least square approach in which every iteration aims at minimizing the total squared error. FISTA introduces in addition a l_1 regularization on the object's coefficients:

$$\min_x \{F(\mathrm{x}) \equiv ||\mathrm{Ax\text{-}b}||^2 + \lambda ||\mathrm{x}||_1\} \quad (31)$$

In FISTA, at every iteration, matrix multiplication is followed by a "shrinkage" in combination with a convergence accelerator ("fast"). At every iterative step, the guessed object's coefficients

are updated according to:

$$\mathrm{x}_{k+1} = T_{\lambda t}\left[\mathrm{x}_k - 2t\mathrm{A}^T(\mathrm{A}\mathrm{x}_k - \mathrm{b})\right] \tag{32}$$

where the shrinkage operator $T_{\lambda t}$ is given by:

$$T_{\lambda t}(\mathrm{x})_i = (|\mathrm{x}| - \lambda t)_+ \mathrm{sgn}(x_i) \tag{33}$$

In layman's terms, shrinkage is equivalent to what a sculptor does by iteratively chipping away small pieces from a large piece of rock. The 'large piece of rock' stands here for our starting guess (x_0) and the 'chisel' is the shrinkage operator. Throughout this paper we use a modified form of the FISTA implementation [4]. FISTA has two tuning parameters: the sparsity degree λ and the step size t. In comparison, Wiener deconvolution has a single one, the signal to noise ratio. For optimal reconstruction, the step size t is maintained as small as possible (10^{-7}) and λ is varied between 0 and 1; 0 is optimal for least sparse objects and 1 for most sparse objects. The optimization is done by comparing the root mean squared error (RMSE) *a posteriori* on reconstructions using different parameters. Since FISTA does not take noise into consideration, background must be subtracted and low pass filtering applied to data before running FISTA.

In order to compare reconstructions by FISTA and Wiener deconvolution, we ran numerical simulations. In Supplementary Fig. 5, three numerical objects were synthesized. Point sources of same amplitude were randomly placed in a $100 \times 100 \times 8$ matrix (considering 8 transverse planes) with various sparsity degrees, so resembling the lysosome vesicles imaged in our experiment. FISTA and Wiener deconvolution were then run based on the computed 2D speckle image (not shown). Here we defined the sparsity coefficient as the ratio between the number of point sources and the number of speckle grains in the volume. With this definition, 1-sparsity-coefficient means that the mean separation distance between point sources is $\lambda/(2NA)$ in each transverse plane (for these simulations, $\lambda/(2NA) = 6.2$ pixels). FISTA can obviously recover objects of a larger range of sparsity than the Wiener deconvolution, even though performances degrades for denser objects. Noteworthy, for dense objects, the algorithm yields better results when the initial guess is close enough to the sought-for object. In Supplementary Fig. 6, the convergence of the two reconstruction algorithms were quantitatively compared by calculating the root mean squared error (RMSE) on the rebuilt 2D speckle image. Results demonstrate that FISTA clearly outperforms Wiener deconvolution and can rebuild objects up to high densities of point sources. Data reconstruction by FISTA required 96.8 seconds to perform 2000 iterations (providing good reconstruction) for a $100 \times 100 \times 10$ object on an intel i5-7500 CPU (3.4 GHz) with 8 GB ram.

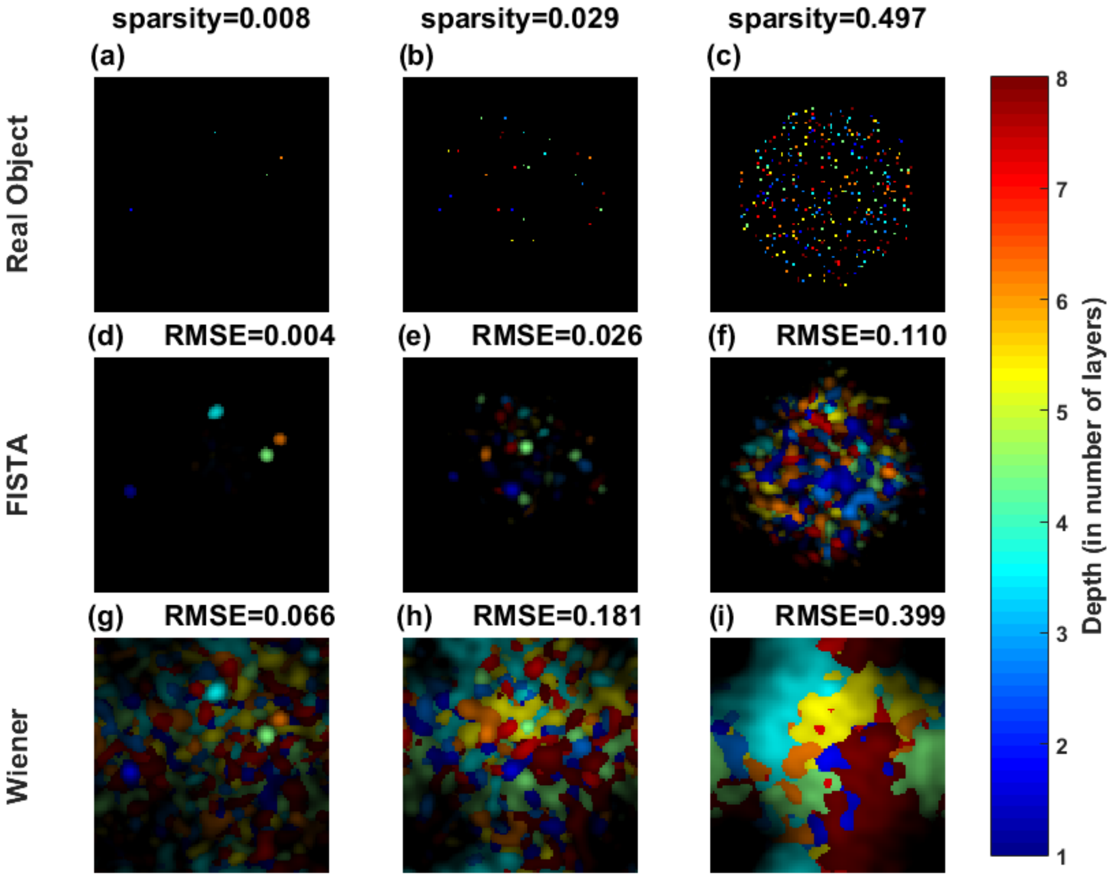

Supplementary Figure 5 | Compressed sensing reconstruction by FISTA (Numerical simulations). Real objects (a)-(c), and rebuilds using FISTA (d)-(f) and Wiener deconvolution (g)-(i). Three different sparsity levels were used: 0.8% ((a),(d),(g)), 2.9% ((b),(e),(h)) and 49.7% ((c),(f),(i)). Sparsity is defined here as the ratio between the point source density and the speckle grains density. Depth information is color-coded.

Supplementary Note 6: Fluorescence signal saturation under short pulse excitation

Modeling of a fluorescent molecule by a two-level system [5], the rate equation of the probability p_1 to be in the first excited state is:

$$\frac{dp_1}{dt} = k_e p_0 - k_f p_1 \tag{34}$$

with $p_0 = 1 - p_1$ the probability to be in the ground state, $k_e = \sigma I(t)/h\nu$ the excitation rate and $k_f = 1/\tau_f$ the fluorescence rate. This differential equation can easily be solved for a step-wise excitation pulse with

$$I(t) = \begin{cases} I_p & \text{for} \quad 0 < t < \tau_p \\ 0 & \text{otherwise} \end{cases} \tag{35}$$

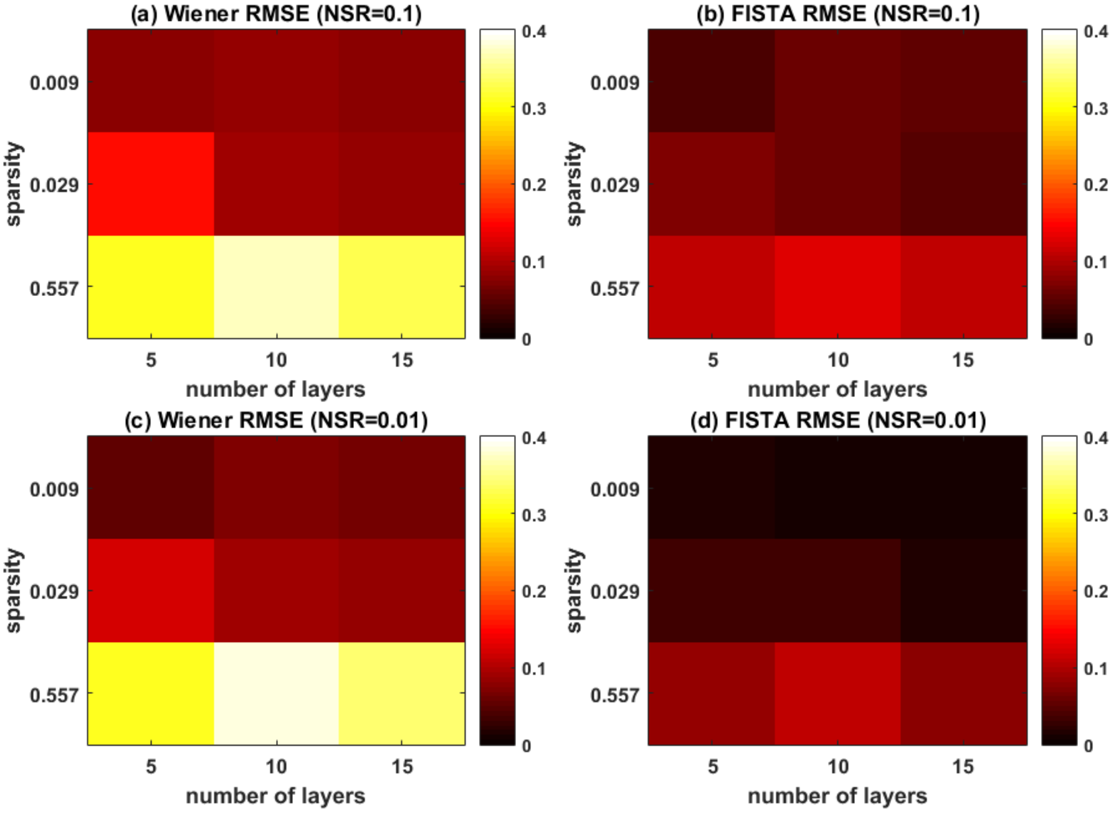

Supplementary Figure 6 | Reconstruction fidelity by FISTA (Numerical simulations). Root mean squared error (RMSE) for reconstructions obtained by (a,c) Wiener deconvolution and (b,d) FISTA; for two different noise to signal ratios: (a,b) $NSR = 0.1$ and (c,d) $NSR = 0.01$. RMSE is computed for different sparsity levels and number of transverse planes.

We then get:

$$p_1(t) = \begin{cases} 0 & \text{for} \quad t < 0 \\ \frac{k_e}{k_e+k_f}\left[1 - e^{-(k_e+k_f)t}\right] & \text{for} \quad 0 < t < \tau_p \\ \frac{k_e}{k_e+k_f}\left[1 - e^{-(k_e+k_f)\tau_p}\right] e^{-k_f t} & \text{for} \quad t > \tau_p \end{cases} \tag{36}$$

Up to a multiplicative constant, the collected fluorescence signal F is:

$$F = \int_{-\infty}^{+\infty} k_f p_1(t) dt \tag{37}$$

$$= \int_{-0}^{\tau_p} k_f p_1(t) dt + \int_{\tau_p}^{+\infty} k_f p_1(t) dt \tag{38}$$

$$= \frac{k_e}{k_e + k_f} \left\{ k_f \tau_p + \frac{k_e}{k_e + k_f} \left[1 - e^{-(k_e+k_f)\tau_p}\right] \right\} \tag{39}$$

Assuming that the pulse width is much shorter than the fluorescence lifetime, the term $k_f \tau_p \ll 1$ can be neglected. Under this assumption, F vanishes for low values of $k_e \tau_p$ and saturates

to 1 under intense illumination conditions, i.e. when $k_e\tau_p \to +\infty$. Under saturated speckle illumination, locations where $k_e\tau_p \ll 1$ only provide a negligible amount of signal and can be neglected. We thus only consider the case $k_e \gg k_f$. Under these two assumptions, Eq. (39) simplifies into:

$$F = 1 - e^{-s} \tag{40}$$

where $s = k_e\tau_p = \frac{\sigma I_p \tau_p}{h\nu}$ may be called the saturation parameter.

Supplementary Note 7: Average fluorescence signal from a speckle pattern

The excitation intensity I_e, and so the saturation parameter s, are is spatially modulated by the speckle pattern. The total fluorescence signal collected when illuminating a uniform fluorescent sample can then be calculated by averaging over the speckle intensity statistics. Assuming a fully developed speckle pattern exhibiting Gaussian statistics, the probability density function of the saturation parameter is given by:

$$\rho(s) = \frac{1}{\langle s \rangle} e^{-s/\langle s \rangle} \tag{41}$$

where $\langle s \rangle$ is the statistical average of s, which, under ergodic assumption is also the spatial average. Thanks to Eq. (40), the average fluorescence signal $\langle F \rangle$ can then be calculated as:

$$\begin{aligned} \langle F \rangle &= \int_0^\infty \rho(s) F(s) ds & (42) \\ &= \frac{1}{\langle s \rangle} e^{-s/\langle s \rangle} \left(1 - e^{-s}\right) ds & (43) \\ &= \frac{\langle s \rangle}{1 + \langle s \rangle} & (44) \end{aligned}$$

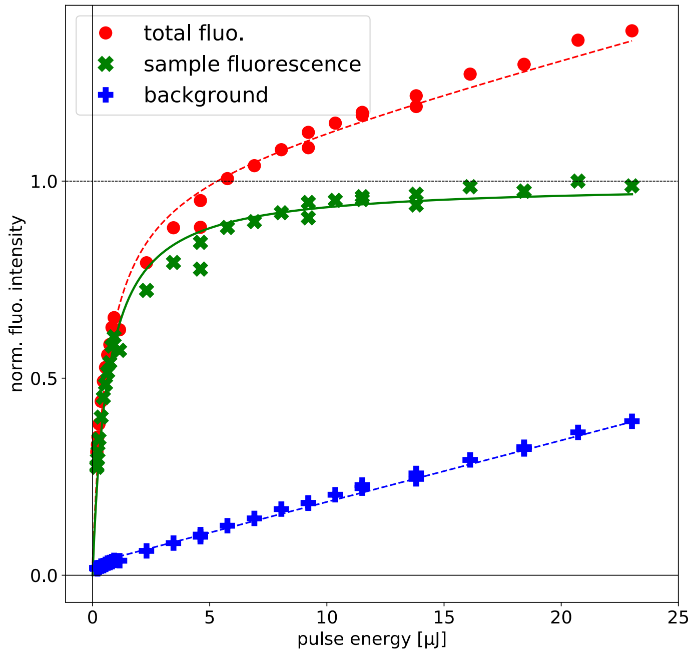

Supplementary Figure 7 | Fluorescence excitation saturation. Characterization of the excitation curve of the fluorescent nano-beads. The raw signal (red circles) contains both the contribution of the bead fluorescence and the background. The latter may be characterized in the absence of fluorescent bead (blue crosses). Subtracting the background to the raw signal gives the excitation curve of the fluorescent nano-bead (green x-crosses). For this experiment, a cluster of fluorescent beads was illuminated with a speckle pattern and the fitting curve thus takes into account the statistics of the intensity distribution.

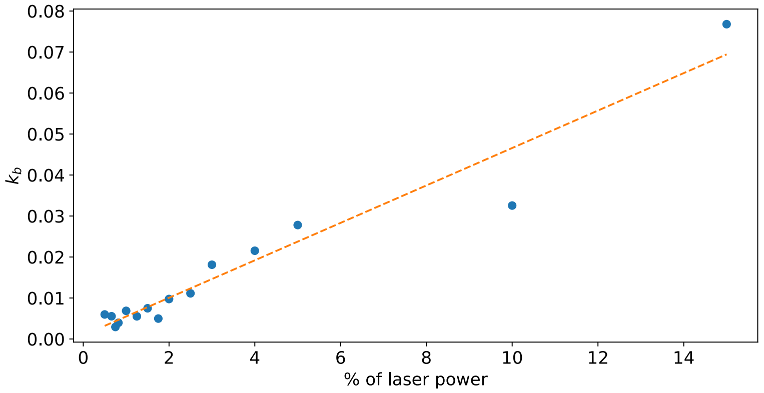

Supplementary Figure 8 | Linear evolution of photo-bleaching rate with laser intensity. Here single fluorescent 100 nm nano-beads were photobleached under illumination by a focused spot of 0.22 NA.

Supplementary Note 8: Axial field modulation at the center of vortices by polarization control

Light being a vector wave, in existing implementation of super-resolution techniques based on the saturation of an optical transition, the pattern is prepared in a specific polarization state [6, 7, 8] in order to yield simultaneous cancellation of all three vector components of the field and to maximize contrast. In purely random waves, the optical vortices of the three vector components are unlikely to overlap, thus preventing the existence of intensity zeros. The axial field amplitude at the center of vortices (of the transverse components) depends on their topological characteristics and on the polarization state of the beam. More in details, vortices in random waves are primarily characterized by their topological charge (± 1), and at first order, can be described by their elliptical intensity profile around the phase singularity [9]. This elliptical profile in intensity is associated with an elliptically non-uniform increase of the phase along the azimuthal coordinate around the phase singularity. Vortices are then described by six parameters [10] whose geometrical ones are the eccentricity of the ellipse and its orientation. The analytical expression and the numerical study of probability density functions of these parameters in random waves have been thoroughly discussed in the literature [10, 11, 12, 9]. To perfectly cancel the axial field at the center of a vortex (of the transverse component), polarization with the same eccentricity and the same axes as the intensity ellipse must be chosen. In a polarized random wavefield, circular polarization thus optimizes the darkness of isotropic vortices of same handedness [13] (exhibiting a uniform phase increase along the azimuthal coordinate) so-ensuring isotropic power-spectrum broadening by optical saturation. An illustration of the anisotropic power spectrum broadening when using linearly polarized light is shown in Supplementary Fig. 10.

In a polarized random wavefield focused with a lens, the vortices of the transverse components coincide. Here, we discuss how the axial field at the center of these vortices depends on the polarization. Without loss of generality, let us choose Cartesian coordinates centered on a given optical vortex and aligned with the main axes of its characteristic ellipse. At first order development, the transverse field may then be written:

$$\mathbf{E}_\perp = \left(\frac{x}{a} + i\sigma\frac{y}{b}\right)\left(\cos\theta \mathbf{e_x} + e^{i\varphi}\sin\theta \mathbf{e_y}\right) \tag{45}$$

where $a, b > 0$ are the semi-minor and semi-major axes of the ellipse, $\sigma = \pm 1$ is the topological charge of the vortex, θ the angle of the polarization ellipse with respect to the x-axis and φ the relative phase between the x and y components of the transverse field. Using the Maxwell-Gauss equation ($\nabla \mathbf{E} = \mathbf{0}$), we obtain the axial field in the paraxial approximation:

$$E_z = \frac{1}{ik}\left(\frac{\cos\theta}{a} + i\sigma e^{i\varphi}\frac{\sin\theta}{b}\right) \tag{46}$$

where k is the wavenumber. The axial field is thus canceled at the vortex center of $\mathbf{E}_\perp$ if the beam is elliptically polarized with the same handedness ($e^{i\varphi} = i\sigma$), the same ellipticity ($\tan\theta = b/a$) and the same orientation as the vortex. Since vortices in random waves contain a broad statistical distribution of ellipticities, intensity cannot be canceled at all vortices at once. The critical role of the polarization state, and thus of the axial field, at high NA and high saturation levels was demonstrated in [13] and is illustrated in Supplementary Fig. 9. Typically, a linearly x-polarized beam minimizes intensity at vortices strongly elongated along the x dimension, and circular polarization minimizes intensity at vortices of same handedness [13]. An illustration of anisotropic power spectrum broadening is shown in Supplementary Fig. 10. For imaging application, optimization of isotropic vortices is preferable in order to obtain isotropic super-resolution in the transverse plane.

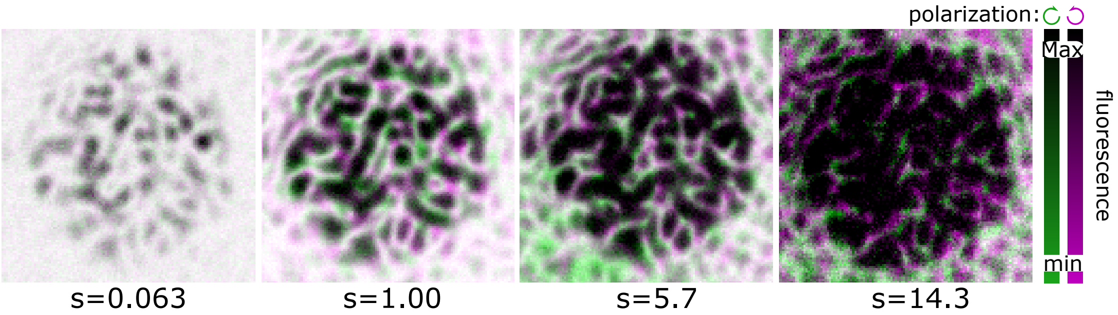

Supplementary Figure 9 | Effect of the axial field on the saturated fluorescence signal. In each image, the green and magenta images are obtained using the same random phase mask on the SLM for excitation, but having right and left-handed circular polarizations, respectively. Changing the handedness of circular polarization mostly modulates the axial field. Here, the contrast of images have been inverted as compared to usual representation of intensities, in order to better visualize the contribution of the axial field. Bright pixels thus code for the dark regions of the speckle which are crucial for super-resolution imaging. From left to right, the saturation parameter s is increased. The significant difference observed between the green and the magenta image observed at large saturation parameters demonstrates the high sensitivity to the axial field. Images taken using a 5 μm speckle spot, with NA $= 0.77$.

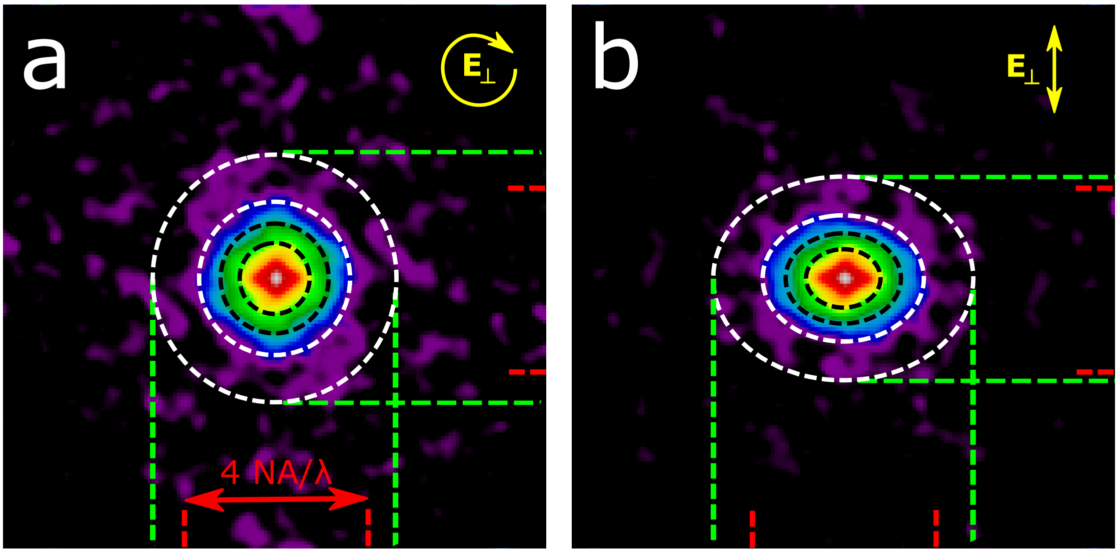

Supplementary Figure 10 | Effect of the polarization state of the speckle pattern on the power spectrum enlargement of the speckle scanning fluorescent image. Circular polarization (a) provides isotropic power-spectrum enlargement while a vertically polarized speckle pattern minimizes the axial field at vortices strongly elongated along the vertical direction, thus enlarging the power spectrum along the horizontal direction (b). Power spectra obtained using NA $= 0.77$ and an average saturation parameter $\langle s \rangle = 1.4$.

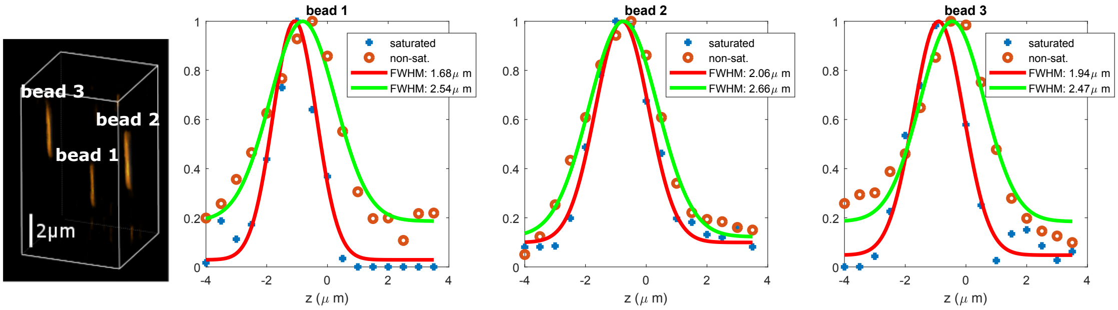

Supplementary Figure 11 | Axial resolution improvement by optical saturation. Axial line profiles of beads labeled from 1 to 3 in the non-saturated regime (red circles) and in the saturated (blue cross) regimes. Full widths at half maxima are mesured by Gaussian fitting (solid lines).

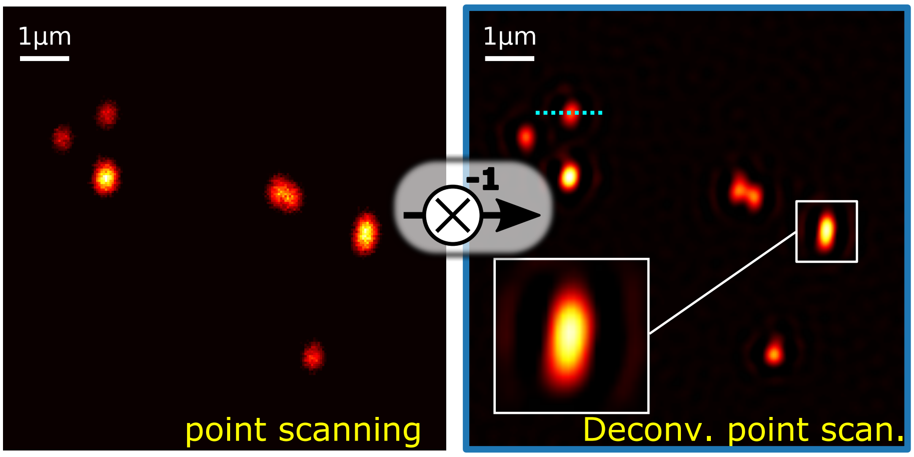

Supplementary Figure 12 | Deconvolved point-scanning image. Point scanning image (left) of the sample shown in Fig. 2f,h,i and k and its corresponding deconvolution (right) by a Gaussian fit of the experimental point spread function.

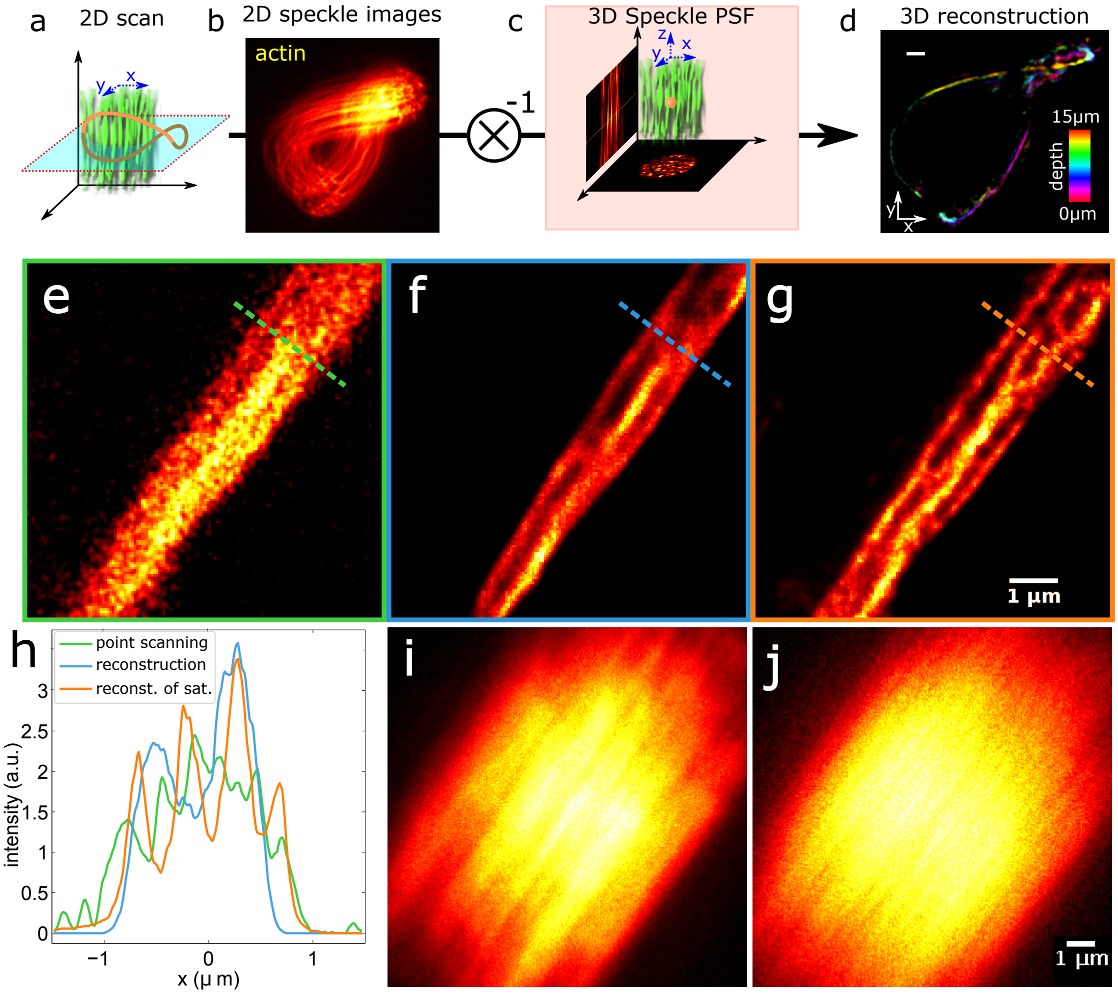

Supplementary Figure 13 | Image of actin filaments. The 2D speckle images of actin filaments are shown in (b). Prior recording of the 3D-SPSF with an isolated fluorescent beads (c) and (Wiener) deconvolution of 2D-scans allows 3D reconstruction of the objects (d). In (d), the axial depth of the 3D-image of the actin filament is color-coded over a 15 μm axial range. Scale bars in (d): 2 μm. Point-scanning image of actin filaments attached on a coverslip (e) and images reconstructed from linear (f) and saturated (g) speckle images. 1000 iterations of an iterative Richardson-Lucy algorithm [14] were run to reconstruct speckle images. Line profiles corresponding to the dotted lines in (e), (f) and (g) are plotted in (h). Raw speckle images corresponding to images (f) and (g) are shown in figures (i) and (j), respectively. All speckle images were recorded using a 10 μm speckle spot with NA = 0.77.

Supplementary References

[1] I. Reed, “On a moment theorem for complex Gaussian processes,” IRE Transactions on Information Theory, **8**(3):194–195 (1962).

[2] Isaac Freund, “Looking through walls and around corners,” Physica A: Statistical Mechanics and its Applications, **168**(1):49 – 65 (1990).

[3] A. Beck and M. Teboulle, “A fast iterative shrinkage-thresholding algorithm for linear inverse problems,” SIAM Journal on Imaging Sciences, **2**(1):183–202 (2009).

[4] Tiep Vu, “FISTA algorithm”, `https://github.com/tiepvupsu/FISTA` (2017).

[5] Jana Humpolickova, Ales Benda, and Joerg Enderlein, “Optical Saturation as a Versatile Tool to Enhance Resolution in Confocal Microscopy”, Biophys. J., **97**(9):2623–2629 (2009).

[6] Xiang Hao, Cuifang Kuang, Tingting Wang, and Xu Liu,
enquoteEffects of polarization on the de-excitation dark focal spot in STED microscopy, J. Opt., **12**(11):115707 (2010)

[7] Silvia Galiani, Benjamin Harke, Giuseppe Vicidomini, Gabriele Lignani, Fabio Benfenati, Alberto Diaspro, and Paolo Bianchini, “Strategies to maximize the performance of a sted microscope”, Opt. Express, **20**(7):7362–7374 (2012).

[8] Bin Yang, Frederic Przybilla, Michael Mestre, Jean-Baptiste Trebbia, and Brahim Lounis, “Large parallelization of STED nanoscopy using optical lattices”, Opt. Express, **22**(5):5581–5589 (2014).

[9] MV Berry and MR Dennis, “Phase singularities in isotropic random waves”, Proc. R. Soc. A-Mat, **456**(2001):2059–2079 (2000).

[10] I Freund, “Optical vortices in Gaussian random wave-fields - statistical probability densities”, J. Opt. Soc. Am. A, **11**(5):1644–1652 (1994).

[11] YY Schechner and J Shamir, “Parameterization and orbital angular momentum of anisotropic dislocations”, J. Opt. Soc. Am. A, **13**(5):967–973 (1996).

[12] I Freund and V Freilikher, “Parameterization of anisotropic vortices”, J. Opt. Soc. Am. A, **14**(8):1902–1910 (1997).

[13] Marco Pascucci, Gilles Tessier, Valentina Emiliani, and Marc Guillon, “Superresolution Imaging of Optical Vortices in a Speckle Pattern”, Phys. Rev. Lett., **116**(9):093904 (2016).

[14] Daniel Sage, Lauréne Donati, Ferréol Soulez, Denis Fortun, Guillaume Schmit, Arne Seitz, Romain Guiet, Cédric Vonesch, and Michael Unser, “Deconvolutionlab2: An open-source software for deconvolution microscopy”, Methods, **115**:28 – 41 (2017).
